# Analysis and Ranking of Protein-Protein Docking Models Using Inter-Residue Contacts and Inter-Molecular Contact Maps

**DOI:** 10.3390/molecules200712045

**Published:** 2015-07-01

**Authors:** Romina Oliva, Edrisse Chermak, Luigi Cavallo

**Affiliations:** 1 Department of Sciences and Technologies, University “Parthenope” of Naples, Centro Direzionale Isola C4, 80143 Naples, Italy; 2 Kaust Catalysis Center, Physical Sciences and Engineering Division, King Abdullah University of Science and Technology, 23955-6900 Thuwal, Saudi Arabia; E-Mails: edrisse.chermak@kaust.edu.sa (E.C.); luigi.cavallo@kaust.edu.sa (L.C.)

**Keywords:** ranking, scoring, analysis, docking decoys, docking models, consensus, contact maps, inter-molecular contacts, protein-protein interactions, structure prediction, interface, COCOMAPS, CONSRANK, CAPRI

## Abstract

In view of the increasing interest both in inhibitors of protein-protein interactions and in protein drugs themselves, analysis of the three-dimensional structure of protein-protein complexes is assuming greater relevance in drug design. In the many cases where an experimental structure is not available, protein-protein docking becomes the method of choice for predicting the arrangement of the complex. However, reliably scoring protein-protein docking poses is still an unsolved problem. As a consequence, the screening of many docking models is usually required in the analysis step, to possibly single out the correct ones. Here, making use of exemplary cases, we review our recently introduced methods for the analysis of protein complex structures and for the scoring of protein docking poses, based on the use of inter-residue contacts and their visualization in inter-molecular contact maps. We also show that the ensemble of tools we developed can be used in the context of rational drug design targeting protein-protein interactions.

## 1. Introduction

Protein-protein interactions are receiving considerable attention as targets for rational drug design. Interest in both inhibitors of protein-protein interactions [1,2,3,4,5,6] and protein drugs themselves [7,8,9,10] is indeed constantly increasing. For these and other applications, structural information, ideally with the availability of the experimental structure of the complex of interest, is invaluable. However, a dramatic disproportion still exists between the number of experimental structures solved for protein complexes and the number of structures available for single proteins [11]. In this scenario, the prediction of a protein-protein complex structure by molecular docking and the scoring of obtained models (topics recently reviewed in [12,13,14,15,16], respectively) will most probably assume a more and more relevant role in drug design.

Details regarding the predicted or experimental structure of a protein complex can be automatically or manually extracted from the 3D model and summarized in tables. However, a simplified representation of the complex interface delivering the relevant information at a single glance is desirable, especially when the comparison between different binding modes is required. With these considerations in mind, we developed a web tool for the analysis and visualization of protein complexes based on the use of intermolecular contact maps, which we named COCOMAPS (COmplexes COntact MAPS) [17]. Intermolecular contact maps are maps where a dot is present at the cross-over of two residues having at least one pair of atoms interacting (*i.e.*, below a cut-off distance). Introduced to provide a more reduced representation of a protein structure than its full 3D atomic coordinates, contact maps have been successfully exploited for describing similarity between protein structures [18]. Analogously, an intermolecular contact map between two or more interacting molecules can identify the surface of interaction uniquely and intuitively. Contact maps represent a sort of fingerprint of the interface and allow one to immediately discriminate between similar and different binding solutions. The advantages of using contact map representations for the alignment of protein-protein interfaces have also been shown [19]. In addition to different types of contact maps, COCOMAPS also provides a complete characterization of the interface in classical terms, *i.e.*, lists of interacting residues (defined on the basis of a cut-off distance), residues at the interface (defined on the basis of the buried surface upon complex formation), intermolecular H-bonds, *etc.*, plus a 3D visualization with interface residues highlighted.

Since contact maps are demonstrated to be a particularly efficient way to represent the interface in protein complexes, we decided to extend this kind of visualization to analyze ensembles of docking models. As an ensemble of models will include both incorrect and hopefully correct solutions, several different contacts will be featured in the ensemble, with different conservation rates (or frequencies). To represent them, we introduced a “consensus contact map”, *i.e.*, a map where the conservation rates of the different contacts are represented on a gray scale (the more conserved the contact, the darker the dot). When measuring the conservation of the various contacts in several ensembles of docking models submitted to CAPRI (Critical Assessment of PRedicted Interactions), a community-wide blind docking experiment, we interestingly found that most conserved contacts in each ensemble usually correspond to the “native” ones, *i.e.*, those observed in the experimental structure [20].

Based on this promising observation, we then developed CONSRANK (CONSensus-RANKing), a consensus approach to the scoring and ranking of docking models, which ranks models based on their ability to match the most conserved contacts in the ensemble to which they belong [21]. Traditionally, scoring functions for protein-protein docking poses rely on two approaches, sometimes combined in hybrid methods. The first approach uses a linear combination of energy terms, while the second approach is statistics-based or “knowledge-based”, as it uses properties derived from experimental structures of protein-protein complexes, usually embodied in atom-atom or residue-residue potentials. CONSRANK thus deeply differs from other valuable algorithms in the field [22,23,24,25,26,27,28,29,30,31,32,33,34,35], as it uses neither knowledge-based nor physics-based energy functions. Application of CONSRANK to the ranking of over 110 targets from different sources showed a very good performance, as it was able to consistently enrich the top ranked positions in correct solutions, provided that they represented an appreciable fraction of the total models [21,36]. It was indeed shown that the performance of CONSRANK is critically dependent on the percentage of correct solutions in the analyzed models ensemble and that failures may be observed when such a percentage is too low. As a general rule, a percentage of correct solutions above 10% was the guarantee of a performance better than random for models obtained from a single docking program, whereas a percentage of 3% was sufficient for the CAPRI models, obtained by different predictors/docking approaches [21,36]. Importantly, CONSRANK was shown to perform significantly better than a simple ligand RMSD-based consensus method [36]. This means that, although the consensus approach clearly has a potential *per se*, the frequency of inter-residue contacts used by CONSRANK is a particularly effective measure to highlight the consensus itself. The CONSRANK algorithm was also shown to perform very well on a limited number of models, provided that more than one correct solution is included in the ensemble, thus extending its applicability to cases where a few dozens of models are available [36].

More recently, we implemented a web server for CONSRANK, which integrates the above analyses, extends them to protein-DNA and protein-RNA complexes and makes them easily available to the scientific community through an advanced interactive web interface (at the URL: https://www.molnac.unisa.it/BioTools/consrank/) [37]. One thousand models with two molecular chains about 150 residues long are processed in 2 min, and interactors made of two chains (e.g., antibodies) can also be dealt with. The CONSRANK server also provides an interactive 3D representation of the consensus map, a “3D consensus map”, where the third dimension is given by the conservation rate of each inter-residue contact. Once the general output has been generated, the user can choose to perform further analyses on single models, allowing one to visualize at a glance how much specific models resemble each other and how well each of them reflects the overall consensus, in terms of inter-residue contacts.

In the following, the potential of the above tools is illustrated by application to exemplary cases, including a possible application in rational drug design targeting protein-protein interactions.

## 2. Intermolecular Contact Maps to Represent the Interface in Protein-Protein Complexes

**Figure 1:**
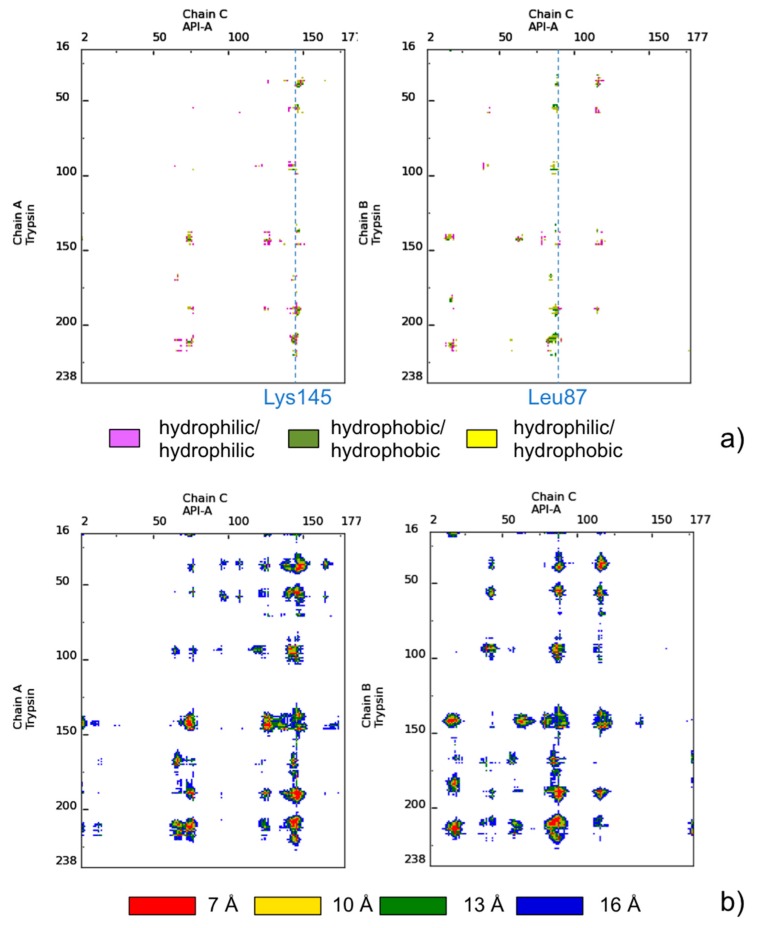
(**a**) COCOMAPS property contact maps and (**b**) distance contact maps for interfaces AC and BC of the trypsin-API-A complex (PDB ID: 3E8L). The color codes are reported in the legends under the maps.

As an exemplary case, we will use in the following a complex between bovine trypsin and the inhibitor API-A from *S. sagittifolia* (PDB ID: 3E8L) [38]. In this complex, the double-headed arrowhead protease inhibitor API-A (chain C) simultaneously binds to two copies of the second protein, trypsin (chains A and B), forming two distinct association modes, corresponding to interfaces AC and BC. These involve the interaction of the trypsin active site with two distinct binding sites on the API-A protein, implicating respectively Lys145 (interface AC) and Leu87 (interface BC) as position P1 (before the scissile bond). Experimental evidence is available for the functionality of both of these binding modes [38,39]. The 3E8L complex is particularly interesting also because it was a target (T40) in CAPRI, Round 18 (see below). In Figure 1, two types of COCOMAPS maps, property and distance range contact maps, are shown for the two interfaces of the complex. COCOMAPS property maps report the contacts colored according to the physicochemical nature of involved residues, while the distance range contact maps report the contacts at increasing distances. From Figure 1, it is immediate that, while trypsin (chain ID: A/B) participates to the binding modes with the same binding site, API-A offers two completely alternative interfaces, centered respectively on the P1 residues Arg145 or Leu87. Further, it is clear that the BC interface (Leu87) is more extended and that, in it, contacts between hydrophobic residues prevail. From the COCOMAPS tables, we see indeed that the BC interface area is 1022 Å^2^ (*vs.* 776 Å^2^ of the AC interface) and that buried residues (more than 90%) are all hydrophobic: besides Leu87 at P1, Ile88 and Phe115.

**Figure 2:**
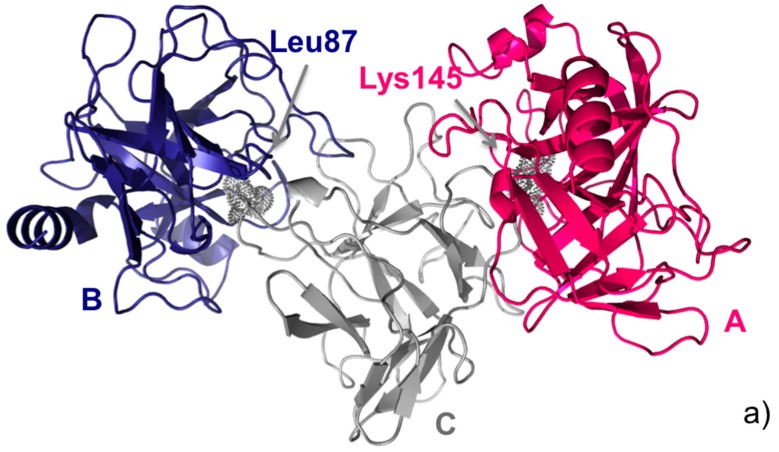

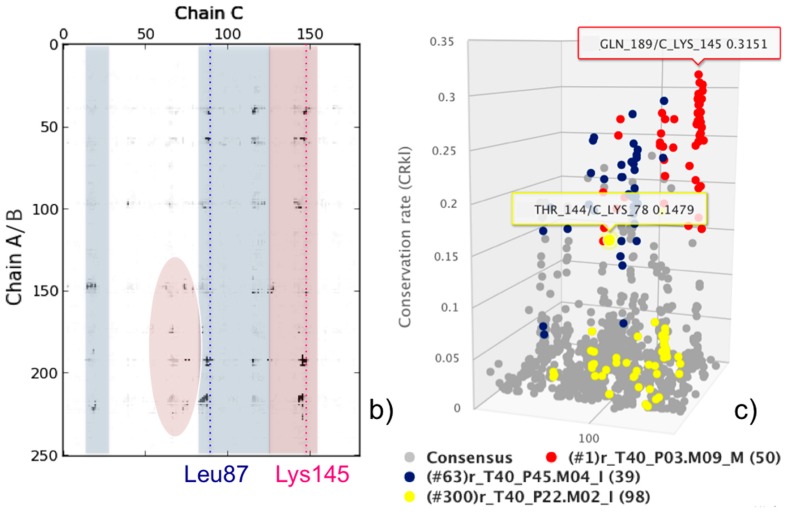
CAPRI target T40. (**a**) Cartoon representation of the trimeric API-A-trypsin complex with the two alternative API-A position P1 residues, Lys145 (interface AC) and Leu87 (interface BC) labelled; (**b**) Consensus map for the ensemble of 368 models submitted for T40 by predictors in CAPRI. Residues corresponding to the two interfaces are highlighted in red and blue, respectively; (**c**) 3D consensus map for the same ensemble of 368 T40 models. The model ranked first (red) corresponds to a correct solution according to the AC interface; the model ranked 63rd (blue) corresponds to a correct solution according to the BC interface; the model ranked 300th (yellow) corresponds to an incorrect solution according to either interface. The contacts consensus is shown in gray. The most frequent contact, between trypsin Lys145 and API-A Gln189, and the native-like contact between trypsin Lys78 and API-A Thr144, also present in the shown incorrect model (yellow), are labelled, and their conservation rate is shown as in the server interactive view.

As mentioned above, this double interface trypsin-inhibitor complex was a target in the CAPRI experiment (Round 18). Therefore, before the experimental structure (shown in Figure 2a) was made available, predictors from all over the world were asked to predict its structure. Predictors submitted a total of 368 models, which were evaluated in CAPRI against both crystallographic binding modes, *i.e.*, T40CA and T40CB. This means that the same models were assessed twice, using as a reference the two crystallographic interfaces, one at a time. Assessment showed that models included 97 and 66 correct solutions, according to the first (Arg145) and the second (Leu87) binding modes, respectively.

In the following, we apply our analysis and ranking approach to this ensemble of 368 models (T40 prediction set). The 2D consensus map from the 368 available models for target T40 is shown in Figure 2b. Analysis of Figure 1 and Figure 2b clearly shows that native contacts corresponding to both interfaces are present in the consensus map. Indeed, CONSRANK top ranks both sets of correct models. However, the consensus corresponding to the CA interface is stronger, and this leads to the top CONSRANK positions being occupied by high-quality models for the CA interface. The 3D consensus map obtained from the CONSRANK server and shown in Figure 2c is particularly illustrative in this case. In the 3D map of Figure 2c, three CAPRI models for T40 are shown. The first two models, ranked first and 63rd by CONSRANK, correspond to two correct models according to the Lys145 (CA interface) and Leu87 (CB interface) binding modes, respectively, and clearly match two different sets of well-conserved contacts. Therefore, CONSRANK is able to highlight contacts characterizing both biological interfaces, which clearly emerge from the background noise. The third model, ranked 300th, is instead incorrect and mostly matches contacts with a very low conservation rate. However, it also matches the well-conserved contact between trypsin Thr144 and API-A Lys78, which is indeed a native one, being present in the corresponding crystal structure at the Leu87 interface. Therefore, analogously to what we observed in several other cases [20,21], even incorrect models can contribute to the conservation of native inter-residue contacts, thus helping to explain the good performance of this scoring approach.

## 3. A Consensus Approach to Analyze and Select Docking Models in a Real-Life Research Case

We recently applied a CONS-COCOMAPS/CONSRANK combined approach to analyze and score the models that we obtained for an antibody-to-antibody complex, related to celiac disease. The two interactors were an anti-type 2 transglutaminase (TG2) antibody, Ab1-MB2.8, derived from celiac patients, and a specific anti-idiotype antibody, Ab2-AIM2, elicited in mouse and competing with TG2 for anti-TG2 binding [40].

**Figure 3:**
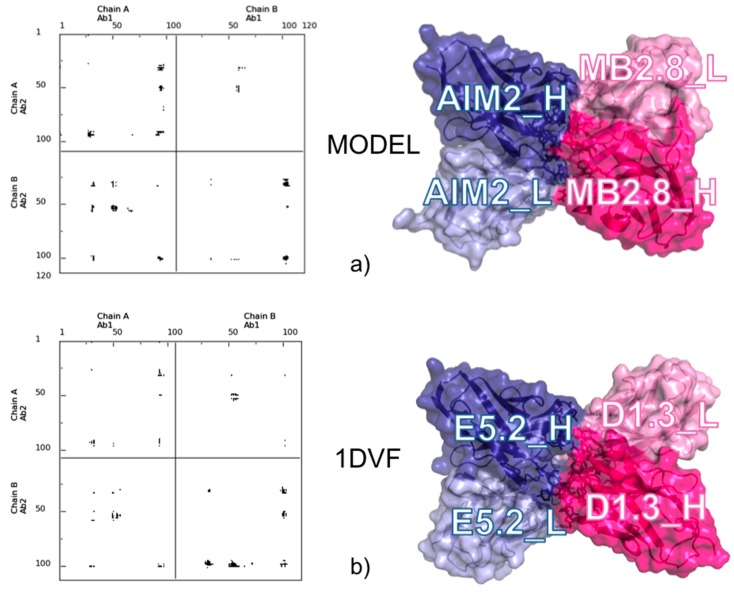
Comparison between the selected docking model for the MB2.8-AIM2 complex and the X-ray structure of the D1.3-E5.2 Ab1-Ab2 complex (1DVF [41]). (**a**) Contact map (**left**) and surface representation (**right**) of MB2.8-AIM2; and (**b**) contact map (**left**) and surface representation (**right**) of D1.3-E5.2. Labels have been added for the Ab1’s and Ab2’s light and heavy chains.

Analysis and selection of the different docking solutions obtained by ClusPro [25] was based on the conservation within them of the inter-residue contacts and relative representation in inter-molecular contact maps. Our approach outlined that the different solutions clearly pointed to a preferred interface area. The model we selected was very well representative of the different solutions found, and this increased our confidence in it. Furthermore, an *a posteriori* comparison between the selected model and the available experimental structures of idiotype-anti-idiotype antibody complexes showed that it is strikingly similar to most of them, and particularly similar to the complex between Ab1-D1.3 and Ab2-E5.2 (PDB ID: 1DVF [41]), despite the obvious differences in sequence. This was immediately clear from comparison of the respective contact maps (see Figure 3) and was then confirmed by detailed analysis of the 3D structures.

## 4. A Dynamic View of the Interface

The approach illustrated above, based on the conservation of inter-residue contacts and their visualization in contact maps, can also be usefully applied for analyzing ensembles of structures other than docking decoys. For instance, it can be extended to usefully characterize the molecular dynamics (MD) evolution of the interface in a protein-protein complex, as well as the interface between domains and sub-domains of a single-chain protein.

**Figure 4:**
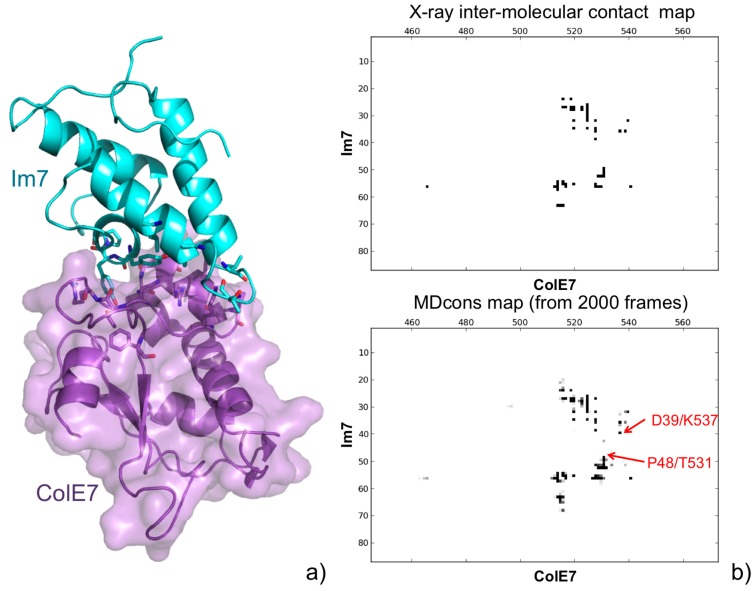
(**a**) A 3D representation of the Im7-ColE7 complex (PDB ID: 7CEI [43]). Residues always maintained during the MD simulations are shown in a stick representation; (**b**) Intermolecular contact map of the Im7-ColE7 X-ray structure obtained by COCOMAPS (top), and consensus map of the 2000 MD snapshots (bottom). Although the interface is quite well conserved, examples of new contacts appearing during the MD simulations are shown (in red) in the consensus map.

We recently introduced a tool specifically devoted to the analysis of the MD trajectories of protein complexes, MDcons [42]. To demonstrate the MDcons potential, we performed MD simulations on two protein complexes, the high-binding affinity Im7-ColE7 structure and the low-affinity CD2-CD58 complex, with interfaces of comparable area and both dominated by hydrophilic residues. To analyze the results of the simulations, we used both classical tools and MDcons. Analysis through the MDcons tool allowed us to outline interesting dynamic features at the interface of both the analyzed complexes, evidencing overall a more rigid interface for Im7-ColE7 and a relatively more flexible and dynamic interface for the CD2-CD58 complex, demonstrating the utility of MDcons in complementing classical analysis tools in the study of the dynamics of protein complexes (see Figure 4 for the Im7-ColE7 complex).

**Figure 5:**
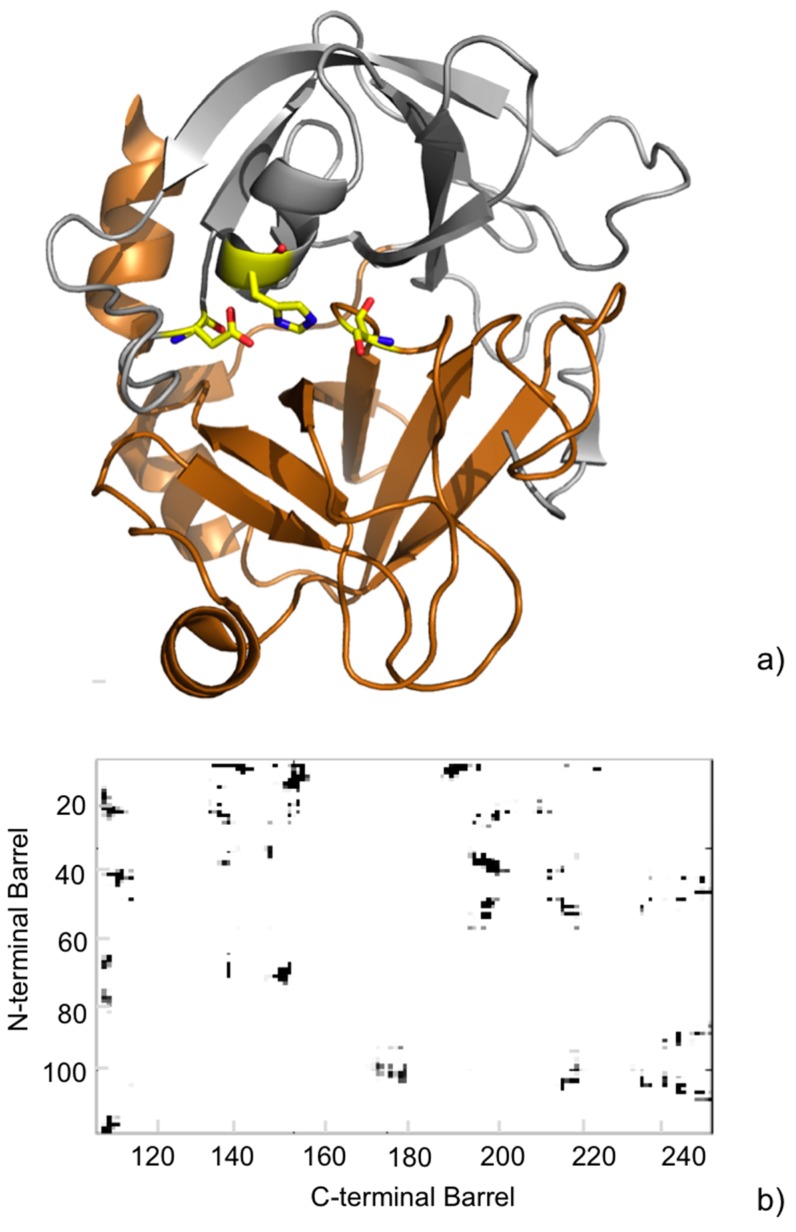
(**a**) Cartoon representation of the crystallographic structure of the catalytic domain of human FXa (PDB ID: 2BOH [45]). The N-terminal β-barrel is oriented up (**gray**) and the C-terminal β-barrel down (**copper**). Residues of the catalytic triad are shown as yellow sticks; (**b**) Inter-barrel consensus contact map from the MD simulations. The observed contacts are colored in a gray scale to indicate their frequency during the MD simulation. The paucity of gray spots in the map indicates a rigidity of the contacts at the interface.

Later, we used the above approach to analyze the MD trajectories for the wild-type (WT) serine protease FXa, playing a major role in the coagulation cascade, and a panel of five pathogenic point mutations [44]. We particularly focused on the protein dynamic behavior at the inter-barrel interface, where the substrate binding site is located. The analyses indicated minor changes for the WT in the contacts at the inter-barrel interface during the simulations (Figure 5). Further, minor differences were observed between the WT and the mutants, indicating that the mutations, although having an impact on the overall flexibility of the protein, have a negligible impact on the core interactions between the two barrels.

Another recent application of the method was the study of the dynamic behavior of the ADAMTS13 Asp173Gly mutant, causing a severe deficiency of the enzyme activity and antigen level, associated with periodic thrombocytopenia and hemolytic anemia in patients. Our approach to the analysis of molecular dynamics simulations allows one to outline that in the mutant, the interface area between two adjacent domains (the metalloprotease and the disintegrin-like domain) significantly decreases during the simulations, while the proline-rich linker region between them undergoes extensive conformational changes [46]. Further, inter-domain contacts are significantly less conserved in the mutant compared to the wild-type enzyme. These findings could be related to the severe observed decrease in the mutant secretion, although the protease was detected inside the cells.

## 5. Assisting Rational Drug Design Targeting Protein-Protein Interactions

**Figure 6:**
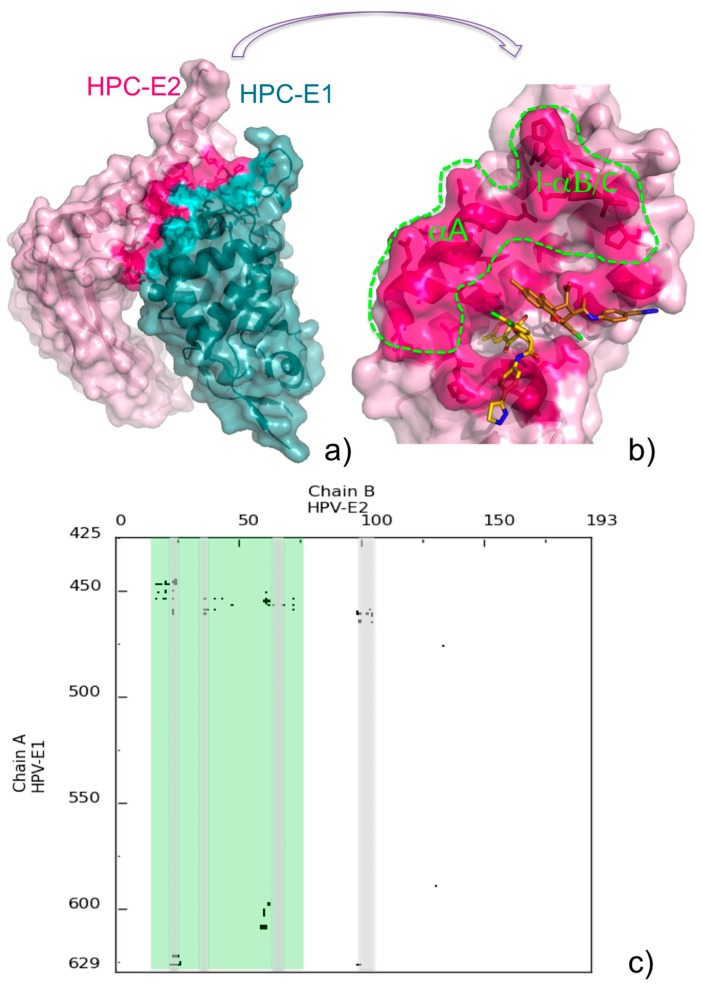
(**a**) COCOMAPS 3D representation of the HPC E1-E2 complex (PDB ID: 1TUE), with interface residues highlighted in teal and hot pink, respectively; (**b**) Inhibitor molecules binding to E2 in the 1R6N complex are shown on the E2 interface colored and displayed as in (**a**). The inhibitor molecules A and B (see text) are colored gold and copper, respectively. The E2 interface untargeted by inhibitors is rimmed with a green dashed line. Labels have been added for the E2 helix A (αA) and the loop between helix B and C (l-αB/C); (**c**) Contact map of the HPC E1-E2 complex. E2 regions contacted by the inhibitors are shadowed in gray, and other regions available for possible inhibitor binding are shadowed in green.

The availability of the three-dimensional structure of a protein-protein complex is key to the possible rational design of inhibitors targeting protein-protein interactions. Deep analysis of the complex interface is in fact the first step toward a successful design. We suggest that COCOMAPS may assist in such analysis, by providing detailed information on the interface, complemented by intuitive and immediate visualization. As a possible example, we show in Figure 6 the complex between the human papillomavirus (HPV) E1 helicase and the transactivation domain of E2 protein (PDB ID: 1TUE, [47]).

The complex is shown in the default 3D representation provided by COCOMAPS (Figure 6a), with residues giving inter-molecular contacts shown in stick and a deeper color, while the contact map for its interface is shown in Figure 6c. The formation of the above complex is essential for the initiation of viral DNA replication. Before the experimental structure of the complex was made available, small halogenated molecules, named “indandiones”, targeting E2 and antagonizing its interaction with E1, both *in vitro* and in cellular assays, were identified by high throughput screening, as potential antivirals for the treatment of Human papillomavirus (HPV) infections [48,49]. The crystal structure of the complex between E2 and one of these inhibitors, specifically BILH 434 (common name, PubChem Compound ID: 5287508) was also reported (PDB ID: 1R6N, [50]). In this structure, two inhibitor molecules were found in the protein binding pocket. The first inhibitor molecule (A) is within 4.5 Å from twelve E2 residues, and its indandione moiety is buried in hydrophobic pocket lines by aromatic residues, while the second molecule (B) only gives weak interactions with E2. Although it possibly constitutes a crystallization artifact, the authors of the structure hypothesized that this second molecule could define additional regions of the E1-E2 interface. The main E2 regions contacted by the inhibitor molecules are shadowed in gray in the map of Figure 6c. In Figure 6b, the inhibitor molecules are displayed on the binding pocket of E2, as in the COCOMAPS representation obtained for the E1-E2 complex. The interface of E2 for the interaction with E1 is colored hot pink. It is immediately clear that not the whole interface is contacted by the inhibitor molecules, which actually only target a small part of it. Although the authors of the E2-inhibitor structure were right in predicting that the second inhibitor molecule was exploring an additional region on the E1-E2 interaction, Figure 6b clearly shows that most of it still remains available for possible inhibitor binding. The E2 region unaffected by the inhibitor binding, rimmed in green in Figure 6b and shadowed in green in the corresponding contact map, involves in particular the C-terminal part of helix A (residues 16–26) and the loop connecting helix B to helix C (residues 61–68). This region could be possibly exploited for the design of more potent inhibitors of the E1-E2 interaction. Besides the intuitive visualization described above, a more detailed characterization of the E1-E2 interface, through the COCOMAPS output tables (number and identity of the residues in contact, interface area, H-bonds at the interface), can offer further guidelines for the rational design of other inhibitors.

Alternatively, when an experimental structure of the protein-protein complex of interest is missing, while structures for the two separate proteins are available, CONSRANK may be used in the analysis of 3D models obtained by docking simulations. In particular, analysis of the conservation of inter-residue contacts provided by the CONSRANK server may be used to help identify possible hotspots for the interaction, to be targeted by designed inhibitors.

## 6. Conclusions and Outlook

Through the examples illustrated above, we have shown that visualization by inter-molecular contact maps is a powerful way to analyze the interface in protein-protein complex structures, providing the needed information in a compact ready-to-read form. Integrated with the measure of the conservation of inter-residue contacts, it also represents a valuable tool to both analyze and rank protein-protein docking models. CONSRANK, the consensus approach we developed based on the above idea, has indeed widely tested and shown to perform competitively with state-of-the-art scoring methods [21,36]. The major drawback of the CONSRANK approach is clearly its critical dependence on the fraction of correct solutions included in the models ensemble to analyze, information that is of course not available during the prediction process. This limitation could be, in our opinion, overcome by the introduction of specific weights for the different contacts, depending on the identity of involved residues. We are now extensively working at deriving such contact-specific weights and modifying consequently the CONSRANK scoring function to improve its performance in disadvantageous cases.

We believe that the approach we reviewed here has great potential, not yet fully exploited, in all applications implying the use of structural information for protein-protein complexes, including drug discovery. As a final remark, we are aware that our approach could usefully be extended to study protein-small ligand complexes, and we are currently working in this direction.

## 7. Technical Details

Within the COCOMAPS, CONSRANK and MDcons analyses, two residues are considered in contact, accordingly to the definition used in the CAPRI experiment [51], if they present at least two heavy atoms separated by ≤5 Å. COCOMAPS classical and property maps were obtained by setting a distance cut-off of 6 Å. The 3D representations of Figure 3 and Figure 6 were obtained by running the .pml file generated by COCOMAPS on PyMol (http://www.pymol.com).
